# The individual-level precision of implicit measures

**DOI:** 10.3758/s13428-025-02873-2

**Published:** 2025-12-08

**Authors:** Jamie Cummins, Ian Hussey

**Affiliations:** 1 Institute of Marketing and Business Administration, University of Bern, Bern, Switzerland; 2 Institute of Psychology, University of Bern, Bern, Switzerland; 3 Bennett Institute of Applied Data Science, University of Oxford, Oxford, UK

**Keywords:** Affect misattribution procedure, Evaluative priming task, Go/No-go association test, Implicit association test, Implicit measures, Measurement precision

## Abstract

Implicit measures are used extensively in psychological science. One fundamental goal of these measures is to provide information diagnostic of an individual’s attitudes or beliefs. After 25 years of research, this goal has not been achieved. We argue that this is because psychologists have not yet even quantified the individual-level precision of implicit measures, much less calibrated them to it. In this paper, we examine the individual-level precision of six different implicit measures across three different attitude domains (race, politics, and self-esteem) using a very large open dataset. Despite some variation, we find that there is substantial room for improvement for the precision of implicit measures as measures of individual attitudes. We recommend that researchers who wish to make theoretical inferences about individuals directly quantify individual-level precision to calibrate their tasks appropriately, both in the context of implicit measures and with tasks in psychological science more broadly.

**Supplementary Information:**

The online version contains supplementary material available at 10.3758/s13428-025-02873-2.

## Introduction

### Implicit measures and individual-level measurement

**Fig. 1:**
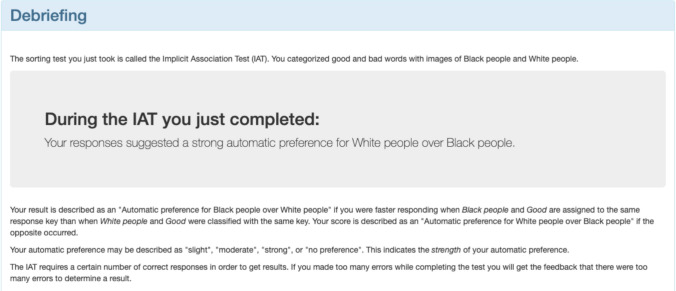
Screenshot of the feedback provided to a participant on the Project Implicit website in January 2023. Notably, since the original publication of our preprint, this feedback has been commendably adjusted to no longer reflect such judgments and provide a more scientifically accurate appraisal. We acknowledge and welcome this change, but retain this figure to provide a sense of context for the reader as to how these scores have historically been interpreted (as well as a sense of the aspirations of these tasks)

Implicit measures are widely used in psychological science and beyond as measures of attitudes, evaluations, and beliefs generally (Greenwald et al., 2022; Kurdi et al., 2019). An often-repeated aspiration for these measures is that they may eventually allow us to make inferences about the attitudes/beliefs of individuals (Fiedler et al., 2006; Greenwald & Banaji, 1995; Greenwald et al., 1998), which is still heavily emphasized in present-day reviews (Greenwald & Lai, 2020). These aspirations are also visible in the public face of these measures; the website Project Implicit (implicit.harvard.edu/implicit) has allowed individuals to complete Implicit Association Tests (IATs) online and receive individual feedback about their level of bias (see Fig. 1; although see also Fig. 1’s caption for important information on how this feedback has changed).

Since feedback about individual-level bias is actively given on the flagship website of the most popular implicit measure, it would be reasonable to assume that meaningful inferences about individual participants’ implicit biases can be made using current methods. Surprisingly and concerningly, this is not the case. In their recent review of meta-analyses, Greenwald and Lai (2020) noted that there have not yet been *any* high-precision implicit measures developed that can make diagnostic claims about (i) the traits of individuals, or (ii) precise trait differences between individuals. Indeed, this is a fundamental barrier: without individually precise measures, we cannot make precise predictions about individuals’ behavior. Despite the long-standing aspirations for individual-level precision, the field has generally made little progress towards this goal. Indeed, it is easier to find examples of attempts to shorten these tasks than to lengthen them (the Brief IAT, Sriram & Greenwald, 2009; shortened Death IAT, Millner et al., 2018). This might make the tasks easier to administer to individuals, but it also makes individuals’ scores less useful for individual predictions (Streiner, 2003).

A significant factor contributing to this stagnation is the lack of direct quantification by researchers of individual-level precision. Although some argue that precision can be improved by enhancing test–retest reliability (Greenwald & Lai, 2020), this alone does not quantify individual-level precision. Scheel (2022) recently argued that many claims in psychological research are “not even wrong”, as they are so underspecified that to be wrong would be an improvement. We would similarly argue that implicit measures are currently ‘not even imprecise’; the field lacks tools to even estimate their precision.

When conducting group-level comparisons, the assertion that (for example) a *given sample* demonstrated “moderate bias” would need to be substantiated not merely by the presentation of a mean score, but by an inference method such as a *p* value or confidence interval. If we consistently applied our otherwise ubiquitous analytic practices to inferences made *in individuals*, we would only say that an individual demonstrated a bias on an IAT if we had reason to reject the null hypothesis that they did not. For example, suppose an individual registers a *D* score of 0.40 on the IAT. Based on the criteria above, they would be given the feedback that they demonstrated a “moderate” bias for White people over Black people. However, if one were to find that the 95% confidence intervals associated with this estimate vary between – 0.10 and 0.90, then the interpretation falters: this score may represent anywhere between “little-to-no bias” and “a strong bias”.

### The standard error of measurement

The field would benefit from the use of an inference method for individuals; one which directly quantifies the measurement (im)precision associated with an individual’s score. Indeed, as some readers will already have noted, quantifying individual-level precision in this manner is well established in literature on psychological assessment, primarily through the use of the standard error of measurement (SEm; Dudek, 1979). The SEm is defined as:

$$\mathrm{SEm}=SD\times \sqrt{1-r}$$

where *SD* refers to the standard deviation, and *r* refers to the test–retest reliability of the measure; 95% confidence intervals can be estimated for an individual’s score as ± (1.96 * SEm). The SEm therefore not only represents a metric of individual-level precision, but also clarifies the precise link between this precision and the group-level property of test–retest reliability.

Despite the general goal of implicit measures researchers to compare individual scores, only two studies to date have used either the SEm or similar metrics to directly estimate individual-level precision in implicit measures. In both cases, these papers assessed the IAT’s precision in the context of racial bias. Schimmack (2021) used a variant SEm (substituting test–retest reliability with measure validity) and found that an IAT *D* score of.30 would have accompanying confidence intervals ranging from – 0.51 to 1.11. Given the bounded nature of the IAT *D* score (from – 2 to 2), this is extremely poor measurement precision. Klein (2020) estimated CIs in terms of individual-level Cohen’s *d* effect sizes (rather than IAT *D* scores), and found a median width of 0.76.

The SEm method is not without its drawbacks. As noted above, the test–retest reliability of the measure is needed to estimate the SEm; however, implicit measures are not monoliths. The test–retest of implicit measures can vary due to a whole host of other features of stimuli and participants (Cummins et al., 2022). Individual participants are also not monoliths. The SEm assumes that the precision of individual scores on a measure will be identical for all individuals; however, it is almost always the case that some individuals’ scores will be better estimated than others (Cummins, 2023; Schmukle, 2023). This reliance on the test–retest statistic leads to assumptions about generalizability at both the domain- and individual-levels that are often not met. On the other hand, estimating the test–retest statistic for different domains or stimuli separately is a potentially laborious and time-consuming process. In principle, the test–retest statistic can be substituted with the internal consistency of split-half reliability of the measure for the estimation of the SEm, which would reduce this burden; however, in this case, the width of the CIs is still assumed to be consistent for all participants, which is likely not the case just as it will not be consistent across domains.

### Bootstrapped confidence intervals for implicit measures

Fortunately, an alternative method can be used, which does not rely on access to test–retest or internal consistency coefficients: namely, by bootstrapping confidence intervals around individual participants’ scores. Bootstrapping, in general, is a statistical procedure that is used to estimate the uncertainty around a given sample estimate (Davison & Hinkley, 1997). The procedure generally involves resampling an existing dataset with replacement many times (e.g., 2000), and a summary statistic is estimated for each bootstrap sample (e.g., the mean). If the sample used for the bootstrapping procedure is relatively representative of the population, then the variation in the means across the bootstrap samples will approximate the sampling distribution of the statistic of interest. This allows researchers to estimate the uncertainty (e.g., confidence intervals) or bias in the original sample statistic without relying on strong parametric assumptions about the underlying population distribution. In the case of implicit measures, we may therefore apply bootstrapping separately to each individual participant’s trial-level data and estimate the confidence intervals around each participant’s score. Since each participant’s data are estimated independently, the width of the confidence intervals of the participant’s scores can therefore vary on an individual basis, allowing for personalized confidence intervals with varying widths between participants, thus providing more information than the more generic SEm approach.

Hussey (2020) previously utilized bootstrapping to estimate implicit measure confidence intervals, specifically around scores on the Implicit Relational Assessment Procedure (IRAP) across 18 different domains. Results were similarly poor as in the above studies using the SEm approach. Researchers have also used this approach to estimate individual-level precision in other contexts, for example, in the assessment of relational reasoning (Cummins, 2023) and inhibitory control (Lee et al., 2025), and a variant in cognitive control (Rouder et al., 2023).

At this point, two facts should be clear: individual-level precision is an important feature of implicit measures, and the limited research which has been done on this matter has been impeded by its methods (Klein, 2020; whose method inappropriately conflated Cohen’s *d* effect sizes with IAT *D* scores), scope of measurement procedures (Schimmack, 2021; Klein, 2020; Hussey, 2020; each of which examined only a single implicit measure) and scope of domains examined (Klein, 2020; Schimmack, 2021; each of which examined the IAT’s precision only in the context of racial bias). A more comprehensive and rigorous investigation into individual-level precision would address a more than 25-year-old problem for one of the most widely employed classes of measures in psychological science.

Using a very large open dataset (Bar-Anan & Nosek, 2014), we investigated the individual-level precision of 6 different implicit measures administered across three distinct domains using the estimation method employed by Hussey (2020). In this preregistered study, we specifically set out to determine (i) how well measures can detect non-zero effects within individuals; (ii) how well measures could discriminate *between individuals*, and (iii) the width of the range of scores that the confidence intervals of individuals’ scores tended to cover. Each of these criteria was chosen based on providing useful information about the performance of the measures on criteria relevant to questions relating to individual precision, which researchers may ask (e.g., how many participants can we say demonstrate bias of any strength in a given direction; how many participants a given participant can be detected as significantly different from).

## Method

### Data source

This study uses openly available data collected on Project Implicit (https://implicit.harvard.edu), originally collected by Bar-Anan and Nosek (2014; data available from osf.io/qf9jx). The data, code, and preregistration for our analyses can be found on the Open Science Framework (osf.io/pq6nf; at the GitHub repository under the “files” tab).

### Sample

The sample used for these analyses was taken from Bar-Anan and Nosek’s (2014) data, collected via the Project Implicit website. A total of 23,413 unique individuals participated in this study (63% women, 36% men, 1% unknown; mean age = 29.1, SD = 12.0). Of this figure, 8.7% completed only one measure, 4.9% completed 2 measures, 7.7% completed three measures, and 31% completed four measures; 45.1% completed more than four measures, of which 10% completed more than ten measures. Detailed information regarding the collection of these data can be found in Bar-Anan and Nosek (2014). The data used in our analytic sample, composed of participants who completed at least one measure in the overall study and met common accuracy and latency performance exclusion criteria (full details in supplementary materials), leading to 21,060 observations in total (i.e., some participants may have completed more than one of the measures). Within this, 6902 participants completed the Implicit Association Test (IAT), 7238 completed the Affect Misattribution Procedure (AMP), 6039 completed the Brief IAT (B-IAT), 6795 completed the Evaluative Priming Task (EPT), 6529 completed the Go-No Go Association Test (GNAT), and 6626 completed the Single-Target IAT (ST-IAT). These completions were divided approximately evenly across the three domains of race, politics, and self-esteem, to which they were assigned randomly within the original study.

In their original study, Bar-Anan and Nosek (2014) also included a seventh implicit measure, the sorting paired-features task (SPF, Bar-Anan et al., 2009). We did not include this task on the basis that it has seen much less use than the other tasks and we were generally unfamiliar with its scoring, in contrast to the other six tasks.

### Measures

For more detailed descriptions, see Bar-Anan and Nosek (2014) and the associated references provided under each measure.

### Implicit Association Test (IAT)

The IAT used in this study followed the procedure outlined in Nosek et al. (2007). A single attitude-object-only practice block of 20 trials was followed by a second practice block of 20 trials involving only evaluative stimuli. The third (20 trials) and fourth (40 trials) blocks involved a combination of the required responses on the two previous blocks. Block 5 was identical to block 1 but with the required response directions switched, and the sixth (20 trials) and seventh (40 trials) blocks incorporated this new configuration in blocks otherwise identical to the third and fourth blocks. The order of required response configurations was randomized between participants.

### Brief Implicit Association Test (B-IAT)

The B-IAT was developed to be a version of the IAT with a shorter administration time and slightly easier instructions for the participant. It requires only two (rather than four) responses on each critical block (Sriram & Greenwald, 2009).

### Single-Target Implicit Association Test (ST-IAT)

The ST-IAT was identical to the IAT but with only one attitude-object (rather than two) investigated on each critical block (Karpinski & Steinman, 2006).

### Affect Misattribution Procedure (AMP)

The AMP followed the procedure described by Payne et al. (2005).

### Go-No Go Association Task (GNAT)

The GNAT here followed the procedure described by Nosek and Banaji (2001), with scores computed based on response latencies.

### Evaluative Priming Task (EPT)

The EPT followed the procedure outlined by Fazio et al. (1995).

### Procedure

For all participants, each session lasted approximately 15 min. Within each session, participants were presented with two “long-duration” and two “short-duration” measures (the implicit measures were divided across these two categories; see Bar-Anan & Nosek, 2014). There were no constraints on participants in terms of the measures they would receive beyond the fact that the same exact measure/domain combination could not be presented twice in one session.

### Research questions

As mentioned above, we addressed three primary research questions in this study.

### RQ1

For each measure, meta-analyzed across domains using multilevel models, what proportion of individual participants’ scores were detectably different from the neutral point of zero effect (i.e., PI = 0.50)? How do these proportions differ between measures?

### RQ2

For each measure, meta-analyzed across domains using multilevel models, what proportion of other participants’ scores were individual participants’ scores detectably different from? In contrast to RQ1, we compared each participant’s score against all other participants’ scores within the same measure and domain. How do these proportions differ between measures?

### RQ3

For each measure, meta-analyzed across domains using multilevel models, what proportion of the observed range of scores did individuals’ 95% confidence interval typically cover? How do these proportions differ between measures?

## Results

### Data processing

#### Scoring algorithm

The implicit measures we compared typically use different methods and metrics for scoring. The IAT, ST-IAT, and B-IAT tend to use a *D* score based on response times; the AMP tends to use proportion of prime-consistent evaluative responses (Payne et al., 2005); the GNAT and EPT tend to be scored based on differential response latencies (alternative scoring approaches have been suggested for the EPT, for example Segal-Gordon & Bar-Anan, 2024; the GNAT can also be scored based on accuracy differentials; Fazio et al., 1995; Gomez et al., 2007; Nosek & Banaji, 2001). These different methods of scoring, and the corresponding differences in scales, score ranges, and error variances associated with them, would limit direct comparisons between the measures. We therefore opted to score every measure using the same analytic method: namely, using probabilistic index (PI) scores (De Schryver & De Neve, 2019). This metric has been referred to by many names, including Ruscio’s A (2008) and the common language effect size (McGraw & Wong, 1992). We refer to it here as the PI on the basis that this is the term used in papers related to the current one and when scoring data from implicit measures (e.g., Hussey, 2020; De Schryver & De Neve, 2019). PI scores estimate the probability of a randomly selected response in one block type being larger (e.g., a longer reaction time or more positive evaluation) than a randomly selected response in the other block type. PI scores also provide a standardized method of scoring data from tasks that are typically derived from different properties of participants’ responses (e.g., accuracy, response times), providing an ideal scoring method to compare multiple measures (see also Cummins et al., 2021). As a probability value, PIs can range from 0 to 1, with the neutral point of zero effect being 0.50 (i.e., equal probability). In this manner, using a single robust and interpretable scoring method allowed for direct comparisons between the measures. Usefully, PI scores nonetheless correlate highly with *D* scores (*r* =.88; De Schryver & De Neve, 2019), which many readers are likely more familiar with. Note that we also generally replicated our findings using the more widely used task-specific scoring methods (see Figures S1 & S2 in the supplementary materials).

#### Confidence intervals around individuals’ scores

Confidence intervals around individuals’ scores were calculated by bootstrapping confidence intervals using the basic (AKA Reverse Percentile Interval; Davison & Hinkley, 1997) method and 2000 resamples. This was implemented in R using the *boot* package (Canty & Ripley, 2021), with the trial-level data of a given participant used as the distribution to be resampled.

## Analyses

### Descriptive statistics

#### PI scores

We first aimed to gauge the modal CI width for each measure across each domain using maximum a posteriori estimation (i.e., computing the mode of the posterior distribution of CI width values). These results are presented in Table 1.

**Table 1:** Maximum a posteriori values for each measure across each domain

Measure	Domain		
	Politics	Race	Self
IAT	0.21	0.21	0.21
B-IAT	0.20	0.20	0.20
ST-IAT	0.17	0.17	0.16
AMP	0.28	0.28	0.28
GNAT	0.19	0.19	0.19
EPT	0.17	0.17	0.17

#### IAT D scores

Although we focus on PI scores in the measures here to make comparisons on the same scale across measures, the inspiration for this work came in part from the criteria associated with the IAT *D* score on Project Implicit, as described in the Introduction. Therefore, as an additional descriptive analysis, we also estimated confidence intervals around the *D* score of the IAT in the context of implicit racial attitudes at each of the cut-offs given by Project Implicit (0, 0.15, 0.35, and 0.65, respectively, for no bias, weak bias, moderate bias, and strong bias). We also provide updated interpretations of these cut-offs in line with the values covered by the associated confidence intervals. These results are presented in Table 2.

**Table 2:** Project Implicit cut-off values for the IAT in the context of racial attitudes, the corresponding confidence intervals, and the updated interpretations based on these confidence intervals

Project implicit cut-off	Interpretation of cut-off	Associated confidence intervals	Appropriate updated interpretation
0	No bias	– 0.38, 0.38	Moderately negative to moderately positive bias
0.15 (weak bias)	Weak bias	– 0.21, 0.51	Weak negative to moderate positive bias
0.35 (moderate bias)	Moderate bias	0.02, 0.68	No bias to strong positive bias
0.65 (strong bias)	Strong bias	0.36, 0.94	Moderate positive bias to strong positive bias

### RQ1. Proportion of effects detectable from zero effect

#### Calculation of scores

**Fig. 2:**
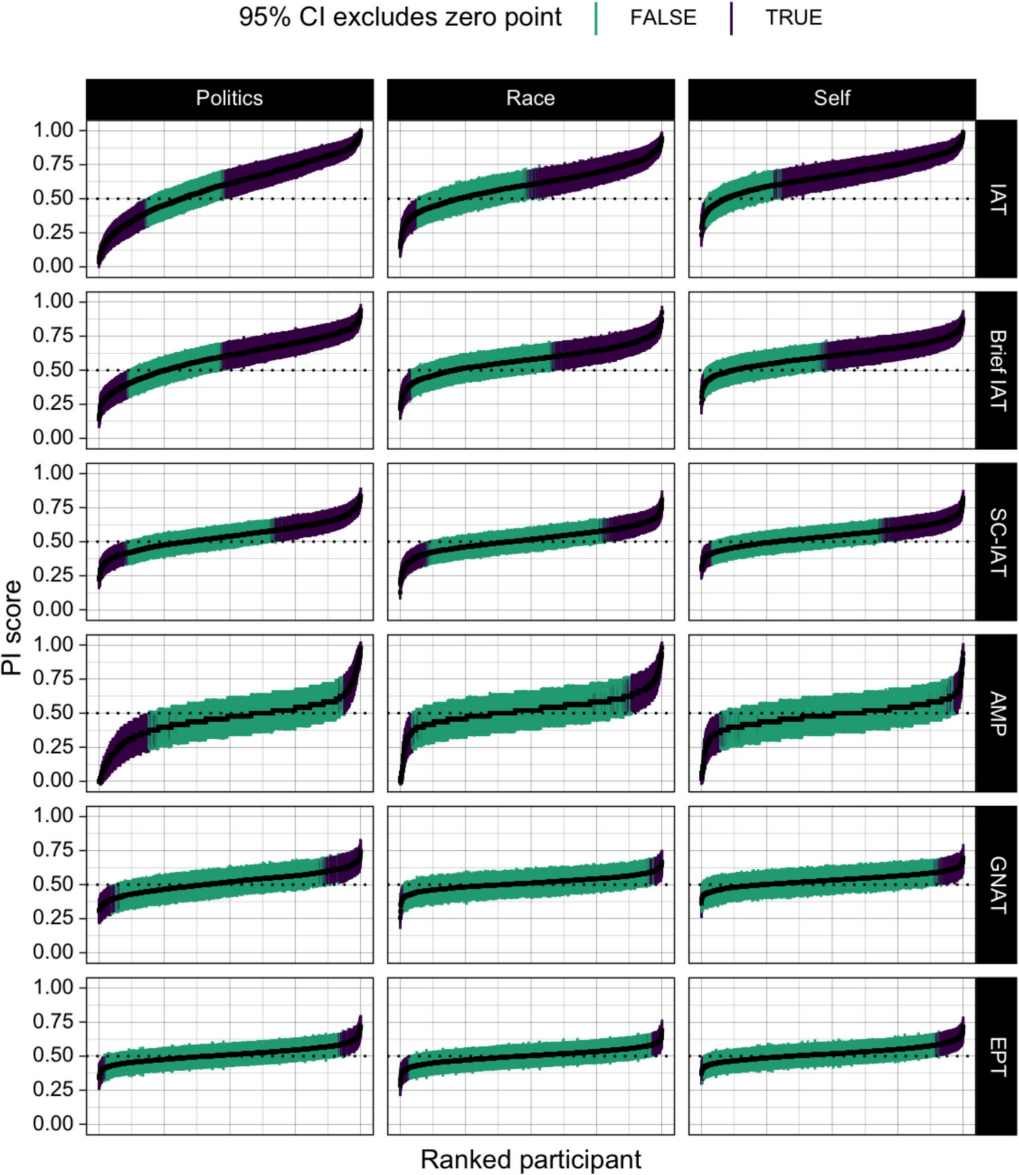
Caterpillar plot of the distribution of PI scores, and their associated confidence intervals, for each participant across each measure and domain. Values marked “TRUE” indicate that confidence intervals excluded 0.5 (i.e., the “zero point” of the PI score), while values marked “FALSE” indicate that confidence intervals did not exclude 0.5. Readers should pay attention to both this binary value as well as the overall width of each CI

The 95% CIs on individuals’ scores were used to assess whether each individual excluded the neutral point of zero effect on the task (i.e., PI = 0.50). Intervals that excluded the neutral point (PI = 0.50) were scored as a detectable effect. A caterpillar plot of individual participants’ scores and their CIs, split by measure and domain, can be found in Fig. 2.

#### Meta-analytic model

To compare the proportions of detectable effects across measures, the data from individuals were meta-analyzed. For each measure and domain, we calculated the proportion of detectable effects and their variance. We then entered the proportions into a linear mixed-effects model using the R package lme4 (Bates et al., 2015). The Wilkinson notation for the model was as follows:

$$proportion\_diff\_zero \sim 1 + measure + \left(1 \left| domain\right.\right), weights = 1/variance$$

**Fig. 3:**
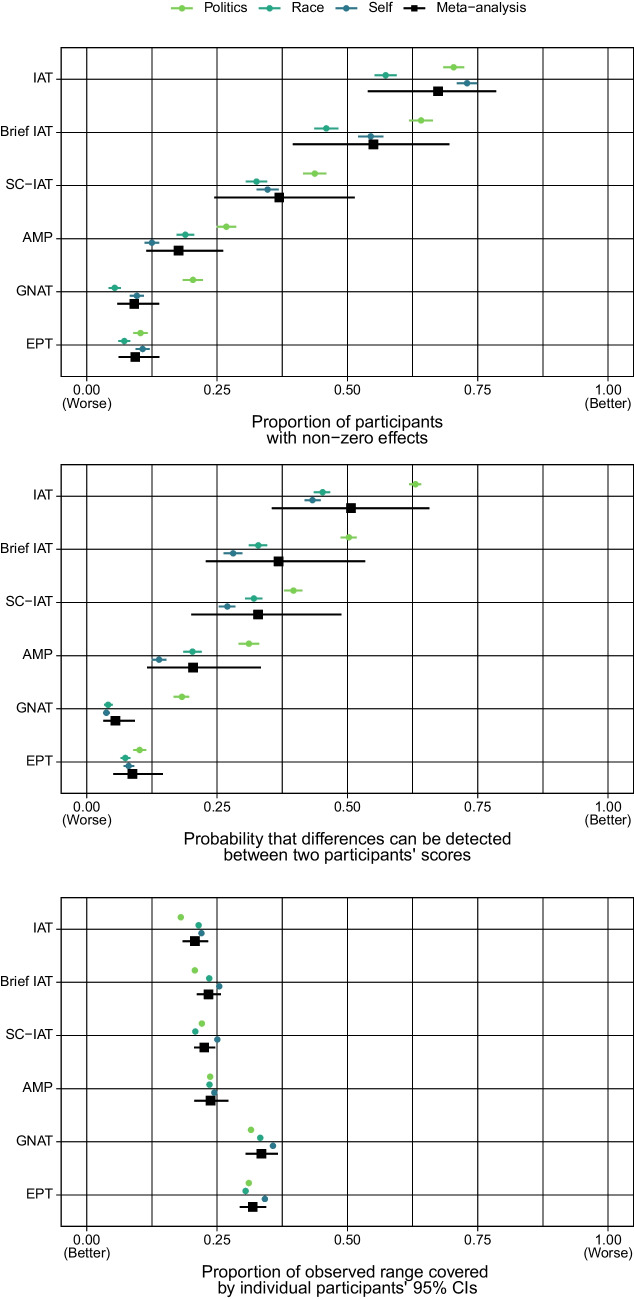
Forest plot for the meta-analytic models associated with the three research questions. The upper third of the plot shows the meta-analytic model for the proportion of participants whose scores differed detectably from zero; the middle third of the plot shows the meta-analytic model for the probability of detectable difference between two participants; and the lower third shows the meta-analytic model for the coverage of the confidence intervals

That is, we entered measure as a fixed effect in order to estimate the proportions for each measure and make inferences about differences between them (i.e., measures are an exhaustive set for our purposes). Domain was entered as a random intercept in order to acknowledge the non-independence of attitudes within each domain, and the fact that there are other domains to be generalized to in principle (i.e., domain is non-exhaustive, and attitude domain is the data-generating signal). We weighted by inverse variance, as is common in meta-analytic models (Viechtbauer, 2005). A forest plot of the individual effect sizes for each domain and the meta-analyzed effect size for each measure can be found in Fig. 3A. Tables containing full results from this and all subsequent models, along with the data presented in the figures in table format, can be found in the online supplementary materials.

Results of the meta-analysis were interpreted with the aid of pairwise comparisons between the measures. These were calculated using the emmeans R package (Lenth, 2022) while also controlling error rates using Holm correction. Results from these pairwise comparisons are presented in Table 3.

**Table 3:** Pairwise comparisons of the estimated marginal means of the proportions of participants discriminable from 0.50 for each measure; a positive value indicates that measure 1 was superior (by that estimated marginal mean value) in the proportion of participants whose scores were detectably different from 0.50 compared to measure 2, whereas a negative value indicates superiority of measure 2 to measure 1 in this respect

Measure 1	Measure 2	Estimated marginal mean difference	95% CIs	*p* value
IAT	B-IAT	0.12	0.03, 0.22	<.001
IAT	ST-IAT	0.30	0.21, 0.39	<.001
IAT	AMP	0.49	0.41, 0.57	<.001
IAT	GNAT	0.57	0.49, 0.64	<.001
IAT	EPT	0.58	0.50, 0.65	<.001
B-IAT	ST-IAT	0.18	0.08, 0.28	<.001
B-IAT	AMP	0.37	0.28, 0.45	<.001
B-IAT	GNAT	0.44	0.36, 0.53	<.001
B-IAT	EPT	0.45	0.37, 0.53	<.001
ST-IAT	AMP	0.19	0.10, 0.27	<.001
ST-IAT	GNAT	0.26	0.18, 0.34	<.001
ST-IAT	EPT	0.27	0.20, 0.35	<.001
AMP	GNAT	0.08	0.01, 0.14	.022
AMP	EPT	0.09	0.02, 0.15	.007
GNAT	EPT	0.01	– 0.05, 0.07	.713

### RQ2. Proportion of scores discriminable from other scores

#### Calculation of scores

We also used 95% CIs on individuals’ scores to assess the proportion of other participants’ scores from which each individual’s score was detectibly different. Pairwise comparisons between each participant and every other participant (separately for each measure and domain) were calculated using the 95% confidence interval on the difference scores between them via bootstrapping, to create one proportion for each participant and its variance. For this and all subsequent analyses, if proportions of 0 or 1 or variances of 0 were obtained, these values were offset by 0.001 in order to allow for meta-analysis.

#### Meta-analytic model

The individual-level proportions were entered into a similar linear mixed-effects model to the previous one:

$$proportion\_discriminable \sim 1 + measure + \left(1 \left| domain\right.\right), weights = 1/variance$$

A forest plot of the individual effect sizes for each domain and the meta-analyzed effect size for each measure can be found in Fig. 3B. Similar to the previous analysis, results from the forest plot were interpreted with the aid of pairwise comparisons between the measures, again using Holm correction. These pairwise comparisons are presented in Table 4.

**Table 4:** Pairwise comparisons of the estimated marginal means of participants who could be discriminated from one another for each measure; a positive value indicates that measure 1 was superior (by that estimated marginal mean value) in the proportion of pairs of participants successfully discriminated compared to measure 2, whereas a negative value indicates superiority of measure 2 to measure 1 in this respect

Measure 1	Measure 2	Estimated marginal mean difference	95% CIs	*p* value
IAT	B-IAT	0.14	0.06, 0.22	<.001
IAT	ST-IAT	0.19	0.11, 0.26	<.001
IAT	AMP	0.30	0.23, 0.38	<.001
IAT	GNAT	0.43	0.38, 0.49	<.001
IAT	EPT	0.42	0.36, 0.48	<.001
B-IAT	ST-IAT	0.05	−0.04, 0.13	.261
B-IAT	AMP	0.17	0.08, 0.25	<.001
B-IAT	GNAT	0.29	0.23, 0.36	<.001
B-IAT	EPT	0.28	0.21, 0.35	<.001
ST-IAT	AMP	0.12	0.03, 0.20	.005
ST-IAT	GNAT	0.25	0.18, 0.31	<.001
ST-IAT	EPT	0.24	0.17, 0.30	<.001
AMP	GNAT	0.13	0.06, 0.20	<.001
AMP	EPT	0.12	0.05, 0.19	<.001
GNAT	EPT	– 0.01	– 0.06, 0.04	.646

### RQ3. Coverage of individuals’ confidence intervals

#### Calculation of scores

We also used 95% CIs on individuals’ scores to assess the typical proportion of the observed range covered by an individual interval. First, the observed interval range was calculated for each domain and measure. Then, each interval was divided by this observed range to calculate a proportion. In order to meta-analyze these proportions, their mean and variance were then calculated.

#### Meta-analytic model

The proportions were entered into a similar linear mixed-effects model to the previous two:

$$ci\_width\_proportion\_mean \sim 1 + measure + \left(1 \left| domain\right.\right), weights = 1/variance$$

A forest plot of the individual effect sizes for each domain and the meta-analyzed effect size for each measure can be found in Fig. 3C. Tables containing the numerical result can be found in the supplementary materials. Results were again interpreted with the aid of pairwise comparisons between the measures using Holm corrections, which can be found in Table 5.

**Table 5:** Pairwise comparisons of the estimated marginal means of the coverage of participants’ CIs for each measure; a positive value indicates that measure 1 was superior (by that estimated marginal mean value) in the coverage of confidence intervals (i.e., they were narrower) compared to measure 2, whereas a negative value indicates superiority of measure 2 to measure 1 in this respect

Measure 1	Measure 2	Estimated marginal mean difference	95% CIs	*p* value
IAT	B-IAT	– 0.03	– 0.05, 0.00	.019
IAT	ST-IAT	– 0.02	– 0.04, 0.00	.073
IAT	AMP	– 0.03	– 0.06, 0.00	.06
IAT	GNAT	– 0.13	– 0.15, – 0.10	<.001
IAT	EPT	– 0.11	– 0.13, – 0.09	<.001
B-IAT	ST-IAT	0.01	– 0.01, 0.02	.274
B-IAT	AMP	0.00	– 0.03, 0.02	.807
B-IAT	GNAT	– 0.10	– 0.12, – 0.08	<.001
B-IAT	EPT	– 0.08	– 0.10, – 0.07	<.001
ST-IAT	AMP	– 0.01	– 0.04, 0.01	.381
ST-IAT	GNAT	– 0.11	– 0.13, – 0.09	<.001
ST-IAT	EPT	– 0.09	– 0.10, – 0.08	<.001
AMP	GNAT	– 0.10	– 0.13, – 0.07	<.001
AMP	EPT	– 0.08	– 0.11, – 0.05	<.001
GNAT	EPT	0.02	0.00, 0.03	.058

## Discussion

A central aim of the implicit measures field has been to use these measures to predict or infer individual participants’ implicit biases. Researchers using implicit measures have been acutely aware that these measures are currently insufficient to do so (Greenwald & Lai, 2020). To date, we have had little sense of exactly how precise these measures are, and no sense of how one measure compares to another. We attempted to unpack this by estimating and comparing the precision of six different implicit measures across three different domains. Our results were stark: all of the implicit measures exhibited rather wide confidence intervals relative to the width of their scales, although some measures (particularly the IAT and its variants) were superior to others. Notably, we also conducted similar analyses on each measure using their native scores; our results were also identical when the native scoring strategies were used compared to the PIs, indicating the robustness of our conclusions (see Fig. S1 and S2 in the supplementary materials).

### Implicit measures should be calibrated for individual-level precision

Given the novel formulation of confidence intervals around implicit measure scores, readers may wonder what exactly an acceptable level of individual-level precision for these measures *would* be to benchmark and interpret the current results. Analogous to questions like “what is the smallest effect size we should care about in a study?” (cf. Anvari & Lakens, 2021), the answer depends on the specific goals and interests of researchers, and we therefore cannot prescribe one-size-fits-all criteria in this regard. However, we can attempt to briefly formulate this based on the implied goals of existing research agendas. Specifically, recall that Project Implicit uses the values of 0, 0.15, 0.35, and 0.65 to denote no bias, small bias, moderate bias, and strong bias, respectively. To minimally infer a significant difference between moderate and strong biases, confidence intervals with widths of 0.6 for the IAT D score would be required; to infer a significant difference between small and moderate biases, CI widths of 0.4 would be required; and to infer a significant difference between no bias and small bias, CI widths of 0.3 would be required. By contrast, the minimal width of CIs around the IAT D score (cf. Table 2) was 0.58 (for “strong” scores); as such, this appears to fall short of the inference goals of Project Implicit. Of course, the PI score was the primary metric of interest in this paper to allow for comparisons between measures; however, given that the IAT was the best-performing measure with the PI, there is certainly substantial room for improvement across the board. It is important to note, however, that these criteria are merely based on one set of benchmarks; the specific desired individual-level precision in each study will be a function of the specific inference goals of the researcher, as with all statistical properties.

In more practical terms, we would recommend that future researchers looking to motivate or identify a desired level of precision begin by asking themselves the question “what level of confidence would I like my individual-level inferences to be at?”. To identify this, one could consider three factors: (1) the minimal differences in “true” scores, without measurement error, that would be considered “different” at the individual level, (2) the estimated individual-level measurement error associated with the measurement instrument, and (3) the level of confidence that these inferences would be desired to be drawn at (e.g., 5% false-positive rate). Factors (1) and (3) are ultimately at the researcher’s discretion and will inherently be specific to their research questions. However, values for (2) can be estimated based on our reported results here. From here, researchers may simulate ground-truth data of known true score differences with individual-level measurement errors and estimate the number of false positives and false negatives when comparing participants’ scores. Researchers may then identify statistical error rates in the individual-level comparisons and determine whether these reach acceptable criteria for their purposes; alternatively, they may also use this approach to identify the level of precision required for their research purposes (Baker et al., 2021).

Researchers may wonder where to start if one were hoping to improve the individual-level precision of an implicit measure. In our view, there are two avenues that could be examined. First, researchers could compare the methodological features of the different implicit measures reported here, given that they exhibit differing levels of precision. For instance, the relative superior precision of the IAT may be attributed to the relatively large number of trials per condition, as well as its use of response times (which may allow for greater inter-individual variability and therefore improve its discriminability between participants, although this will necessarily also be task design- and scoring-dependent). Similarly, the IAT provides practice blocks to participants (which may aid in reducing random measurement error) while still providing a relatively challenging, speeded-response context in critical trials. This relatively challenging response context may similarly enhance inter-individual discriminability.

Second, researchers may look to more general knowledge relating to psychometrics (e.g., test–retest reliability, internal consistency) to improve the individual-level precision of these measures. The most brute force approach is to simply increase the number of trials: by definition, the precision of an estimate will improve with more observations. More sophisticated methods may also be considered. For instance, ensuring the unidimensionality of stimulus items for a given attribute or category would reduce measurement error, in turn improving individual-level precision (cf. Reise et al., 2013). By extension, stimulus items could be better vetted to ensure the absence of issues relating to differential item functioning (i.e., where responses to a particular stimulus item from individuals with the same level of bias from different demographic groups are affected by factors external to the bias itself; Zumbo, 1999). Methods from item response theory (IRT) have been developed specifically to assess factors such as unidimensionality and differential item functioning and may be of utility to implicit measures researchers attempting to improve individual-level precision (Hambleton et al., 1991).

A previous reviewer of this manuscript took objection to the labelling more/less precision in scores as “better” and “worse” (cf. Figure 3), arguing that there will be cases in which, for example, researchers would not expect perfect discriminability between all participants, as some participants may have the same level of true bias for a construct. However, such a position does not mean having a more precise measurement instrument is “worse”; rather, it simply means that the minimal level of measurement precision required in a research project will depend on the inferential goals of the researcher. Analogously, suppose a researcher wished to compare the temperature of two objects and had two thermometers available to them: one that gave readings precise to the tenth decimal of a degree, and one that gave readings precise to the first decimal of a degree. If the researcher wished to compare objects with an expected temperature difference of 5 degrees, then clearly the less precise thermometer would suffice (although the more precise thermometer could also be used for this purpose). However, if the expected temperature difference was in the range of 5 decimal places, then only the more precise thermometer would be more appropriate for use. More critically, however, the more precise thermometer is psychometrically superior in all cases, independent of the researcher’s specific question.

While these comparative assessments of the individual utility of six common implicit measures are useful in and of themselves, the most important aspect of this work is that it provides researchers with a framework for assessing the precision of their implicit measures. This has until now been sorely lacking in the implicit social cognition literature. Researchers may now have a sense of how precisely estimated individual scores on implicit measures are. In the context of *D* scores in the IAT as highlighted in Table 2, scores of 0 in the IAT can indicate anywhere between moderate negative and moderate positive bias, rather than no bias (as was long-stated on the Project Implicit website), and it is only at a score of around 0.35 that one can reliably conclude that that individual has a non-zero bias (and even then, this bias may barely differ from zero).

This work more generally highlights the importance of a detailed focus on measurement within implicit measures research—a need that is echoed throughout psychological science (Flake & Fried, 2020; Hussey & Hughes, 2020). Whereas the goal of individual-level prediction has been present in the field of implicit measures for 25 years, directly estimating and measuring this has been neglected. Indeed, the disconnection between our stated goals and measurement practices is alarming. We hope this can serve as an illustration for other research domains with similar issues; if we aspire to certain goals, we must be able to quantify whether those goals are being achieved.

### Individual-level precision beyond implicit measures

Many other fields of psychology aim to make claims about individuals without estimating individual-level effects. McManus et al. (2023) recently noted that the majority of psychological researchers wish to make claims about at least a majority of individuals when conducting experiments. Others have proposed that the presence or absence of effects within individual participants represents a more meaningful effect size metric than group-level approaches (Grice et al., 2020). The use of bootstrapping for individual-level estimation can be applied robustly across research areas; it can be done with any performance-based task that consists of response times and/or accuracy scores. This method also allows for individualized confidence intervals, rather than the application of CIs of a generic range across all participants (as would be the case if the CIs were derived from test–retest or internal consistency statistics using the standard error of measurement). If the goal of using a task is to make individual-level inferences, then researchers should strongly consider quantifying individual-level precision as early as possible in the process of developing their measure.

### Implicit measures beyond individual-level precision

Our precision analyses here are fitted to the empirical data of the implicit measures; however, there may also be utility or interest in applying such methods of precision to the *process* level, particularly if researchers expect that multiple distinct processes produce implicit measure scores (e.g., in the context of QUAD modeling; Conrey et al., 2005). In principle, the application of precision analyses to process-level models should also be possible using bootstrapping. Such an approach would allow confidence intervals to be fitted to the parameter estimates from these models, which in turn would give a sense of the uncertainty around the estimates of contributions from each of the separately modeled processes. Indeed, this would be particularly useful in cases where researchers wish to make inferences about differences in the extent of influence of different processes within a given participant.

While our primary focus was on estimating individual-level precision within each task, future research could examine how precision relates to rank-order consistency across tasks measuring the same domain. For example, if two tasks both assess implicit racial attitudes, the more precise task should yield scores that better align with participants’ rankings on other conceptually related measures. This cross-measure comparison would provide useful additional data for evaluating convergent validity as a function of precision.

It is important to note, however, that there remain conceptual issues with the use of implicit measures that cannot be addressed by improving individual-level precision. For instance, even if an implicit measure were to be exceptionally individually precise, this says little about *what construct* is being precisely measured. As one reviewer argued, “implicit measures” are not a particularly meaningful theoretical or empirical set, given that they are typically weakly correlated at best and that their primary unifying feature is methodological (i.e., indirect measurement) rather than theoretical (Corneille & Hütter, 2020). We strongly agree, and we consider the “implicit measures” to likely represent an instance of the jingle fallacy: namely, the assumption that multiple measures capture the same construct because they share the same name (Kelley, 1927). Of course, we also recognize that this perspective is not universally shared among researchers. Critically, questions relating to individual-level precision cannot address these more fundamental issues, which are present in the field. For one of the core stated aims of the field (namely, identification of individual attitudes/evaluations), individual-level precision represents a necessary but not sufficient step to achieve this.

### Limitations

A limit on the generalizability of our findings relates to our selection of measures. The tasks examined here may not be representative of all implicit measures. Even within those we tested, our results may not generalize to those measures in all contexts; we investigated the properties of the measures across three domains, but countless others may lead to differences in the psychometric properties of the tasks. We strongly advocate testing the generalizability of these results across other implicit measures, domains, and psychological tasks in future research.

Although a powerful approach to estimation, bootstrapping is also not without its limitations. For procedures with a limited number of trials, these approaches may produce biased estimates (Mostofian & Zuckerman, 2019). Although other bootstrapping methods exist that can correct for bias due to small samples (e.g., bias-corrected and accelerated bootstrapping; Puth et al., 2015), these methods can suffer from convergence issues or may produce scores in some bootstrap samples that fall outside of the possible bounds of the scale (e.g., outside of 0 and 1 in the PI). Although our findings were relatively robust across different bootstrapping methods, it is critical to consider the method of choice when using this approach carefully.

One reviewer questioned whether data collected online (vs. lab-based collection) may influence individual-level precision. We would suggest that existing research seems to indicate that online data collection tends to produce data of roughly the same quality and psychometric properties as in-lab data collection (e.g., McConnell et al., 2025). Additionally, it is certainly the norm in modern implicit measures research to collect data via online samples; indeed, the data analyzed here were from Project Implicit, the largest source of data on implicit measures in the field. However, future research could certainly examine in more depth whether individual-level precision varies explicitly as a function of online vs. in-person data collection methods.

## Conclusion

This work represents the first comparison of multiple implicit measures in terms of their individual-level measurement precision. Although we hope that our results will be informative and useful to researchers who have used, are using, or will use implicit measures in their research, our ultimate hope is that psychological researchers *in general* will explicitly use metrics of individual-level precision as benchmarks to improve their tasks where applicable. Psychological science cannot be a science of persons without the precise measurement of persons.

## Supplementary Information

Below is the link to the electronic supplementary material.

Supplementary file1 (PDF 849 kb)

## Data Availability

We did not conduct the original study or data collection. However, the study materials and data were made openly available by the original authors; a copy of the relevant materials can be found here (https://osf.io/3n8yv); all primary data from the original study are also publicly available (https://osf.io/uqrbn). **Code availability:** All analysis scripts are publicly available (https://osf.io/p4bnh).

## Declarations

### Preregistration information

The hypotheses and analysis plan/code were preregistered (https://osf.io/qk9ar) on 31/08/2022, prior to the commencement of the analysis of the data. There were minor deviations from the preregistration (for details see Supplementary Table S1).
