# Supplementary material for: The individual-level precision of implicit measures
Source: Behav Res Methods. 2025 Dec 8;58(1):21. doi: 10.3758/s13428-025-02873-2 (PMC12686091; doi:10.3758/s13428-025-02873-2)
Supplement: Supplementary file 1 — Supplementary file1 (PDF 849 kb) [file 13428_2025_2873_MOESM1_ESM.pdf]

Comparing the suitability of 6 implicit measures for individual use

Supplementary Materials

For Review Only

Analyses conducted on native scores of the implicit measures

Two separate reviewers noted that it may be informative to examine not only the performance of the measures using the PI scores, but also when using their native scores, given that it may be the case for some measures that they are more performant with their native scores than with the PI. We therefore provide Figures S1 and S2, which correspond to Figures 2 and 3 in the manuscript respectively, but where the measures are scored using their native scoring rather than the PI. As can be seen, the pattern of results is essentially the same as for when the PI is used.

For Review Only

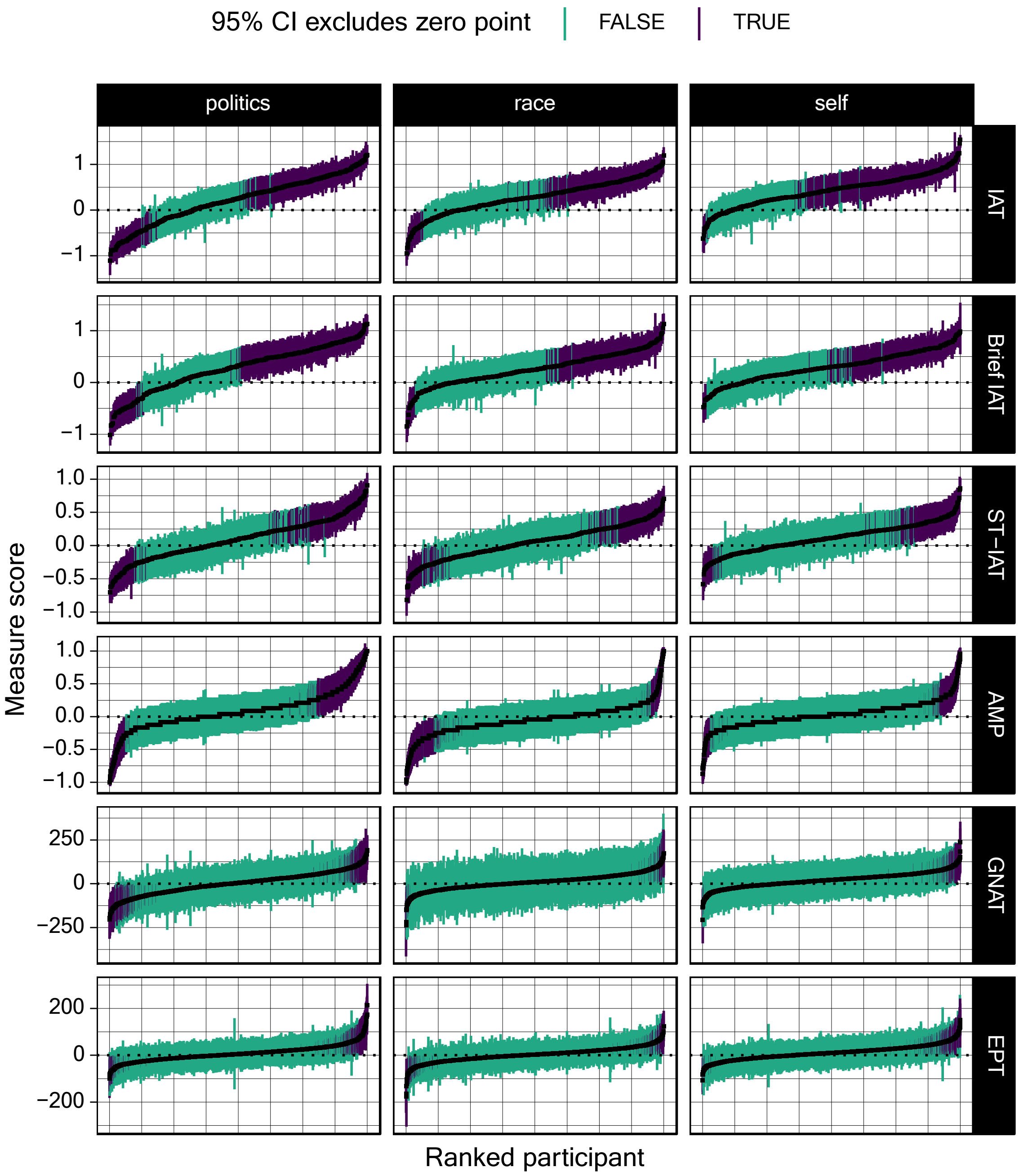

Figure S1. Caterpillar plot of the distribution of the native scores of each measure, and their associated confidence intervals, for each participant across each measure and domain.

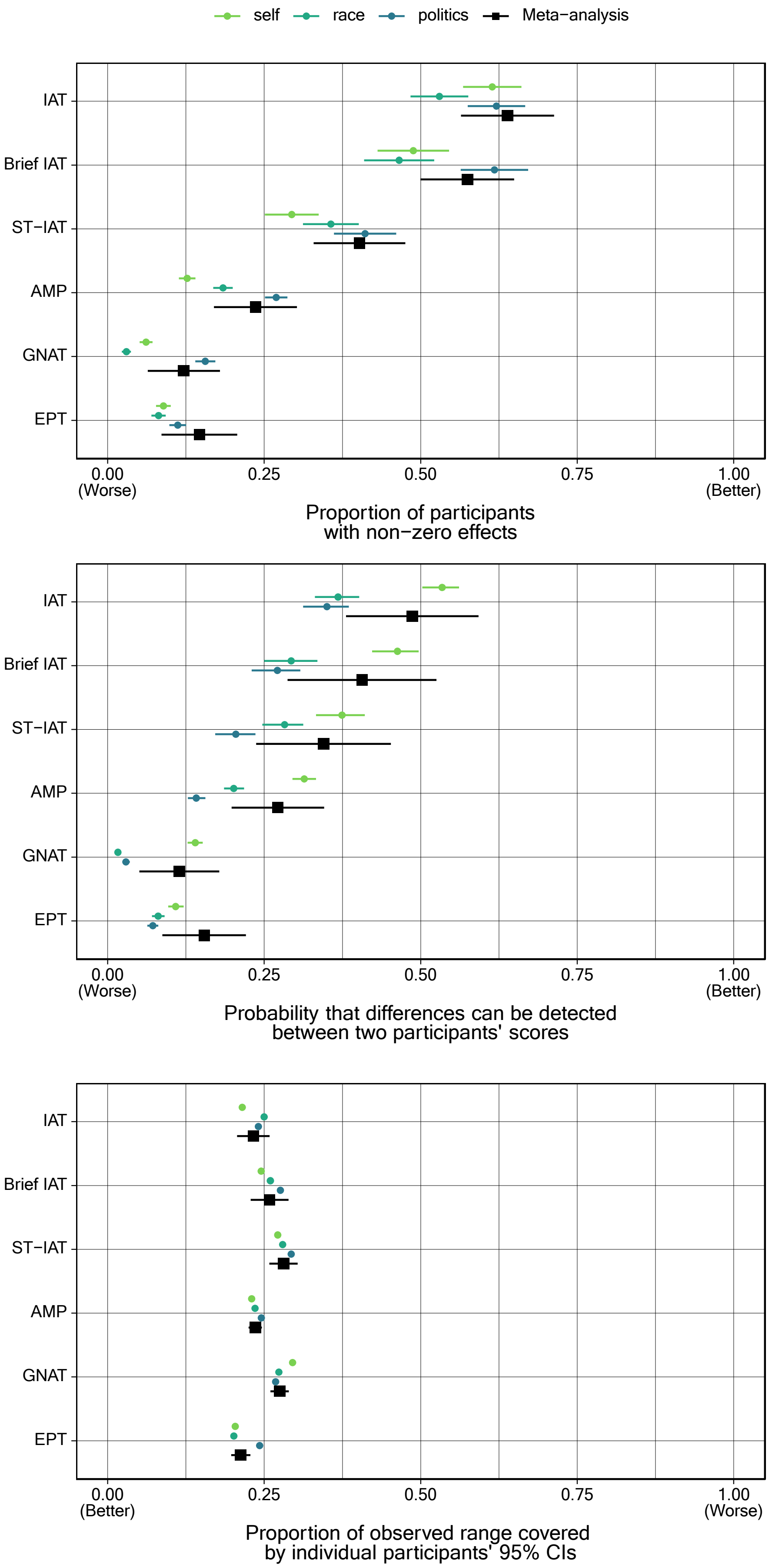

self
race
politics
Meta-analysis
IAT
Brief IAT
ST-IAT
AMP
GNAT
EPT
0.00 (Worse)
0.25
0.50
0.75
1.00 (Better)
Proportion of participants with non-zero effects
Probability that differences can be detected between two participants' scores
0.00 (Better)
1.00 (Worse)
Proportion of observed range covered by individual participants' 95% CIs

Figure S2. Forest plot for the meta-analytic models associated with the three research questions using the native scores for each measure. The upper third of the plot shows the meta-analytic model for the proportion of participants whose scores differed detectably from zero; the middle third of the plot shows the meta-analytic model for the probability of detectable difference between two participants; and the lower third shows the meta-analytic model for the coverage of the confidence intervals.

For Review Only

Elaborated information on in-text analyses

RQ1

Plot data

Table S1 provides the numerical information plotted in Figure 1 in the paper.

Table S1. Data from Figure 1 in the manuscript.

measure	estimate	ci_lower	ci_upper	pi_lower	pi_upper
IAT	0.67	0.59	0.75	0.55	0.79
Brief IAT	0.55	0.46	0.64	0.43	0.67
SC-IAT	0.37	0.29	0.45	0.25	0.49
AMP	0.18	0.11	0.25	0.07	0.30
GNAT	0.11	0.04	0.17	0.00	0.22
EPT	0.10	0.03	0.16	-0.01	0.20

w Only

RQ2

Plot data

Table S2 provides the numerical information plotted in Figure 2 in the paper.

Table S2. Data from Figure 2 in the manuscript.

measure	estimate	ci_lower	ci_upper	pi_lower	pi_upper
IAT	0.52	0.42	0.61	0.39	0.64
Brief IAT	0.38	0.28	0.48	0.25	0.51
SC-IAT	0.33	0.23	0.43	0.20	0.46
AMP	0.21	0.11	0.31	0.08	0.34

measure	estimate	ci_lower	ci_upper	pi_lower	pi_upper
GNAT	0.08	0.00	0.17	-0.04	0.20
EPT	0.09	0.01	0.18	-0.03	0.22

RQ2

Plot data

Table S3 provides the numerical information plotted in Figure 3 in the paper.

Table S3. Data from Figure 3 in the manuscript.

measure	estimate	ci_lower	ci_upper	pi_lower	pi_upper
IAT	0.21	0.18	0.24	0.12	0.30
Brief IAT	0.23	0.21	0.26	0.14	0.32
SC-IAT	0.23	0.20	0.25	0.14	0.32
AMP	0.24	0.20	0.27	0.14	0.33
GNAT	0.34	0.31	0.36	0.24	0.43
EPT	0.32	0.30	0.34	0.23	0.41

Unreported logit-transformed linear mixed-model analyses

As mentioned in the deviations from preregistration document, we had originally preregistered that we would model our data using logit-transformed linear mixed-effects models before realising that modelling the untransformed proportion data would be both more appropriate (based on simulations from Kubinec, 2023) and more interpretable. For transparency, we report the results from the originally preregistered analyses below.

RQ1. Proportion of effects detectable from zero effect

Meta-analytic model

In order to compare the proportion of detectable effects between measures, the data from individuals was logit transformed and meta-analyzed. For each measure and domain, we calculated the proportion of detectable effects and its variance. We then entered the proportions into a linear mixed-effects model using the R package lme4. The Wilkinson notation for the model was as follows:

proportion_diff_zero_logit ~ 1 + measure + (1 | domain), weights = 1/variance

That is, we entered measure as a fixed effect in order to estimate the logit-transformed proportions for each measure and make inferences about differences between them (i.e., measures are an exhaustive set for our purposes). Domain was entered as a random intercept in order to acknowledge the non-independence of attitudes within each domain, and the fact that there are other domains to be generalized to in principle (i.e., domain is non-exhaustive, and attitude domain is the data generating signal). We weighted by inverse variance, as is common in meta-analytic models. A forest plot of the individual effect sizes for each domain and the meta-analyzed effect size for each measure can be found in Figure S. Table S4 provides the numeric values which are plotted is Figure S3. Table S5 provides the pairwise comparisons for the estimated marginal means of each measure for RQ1.

For Review Only

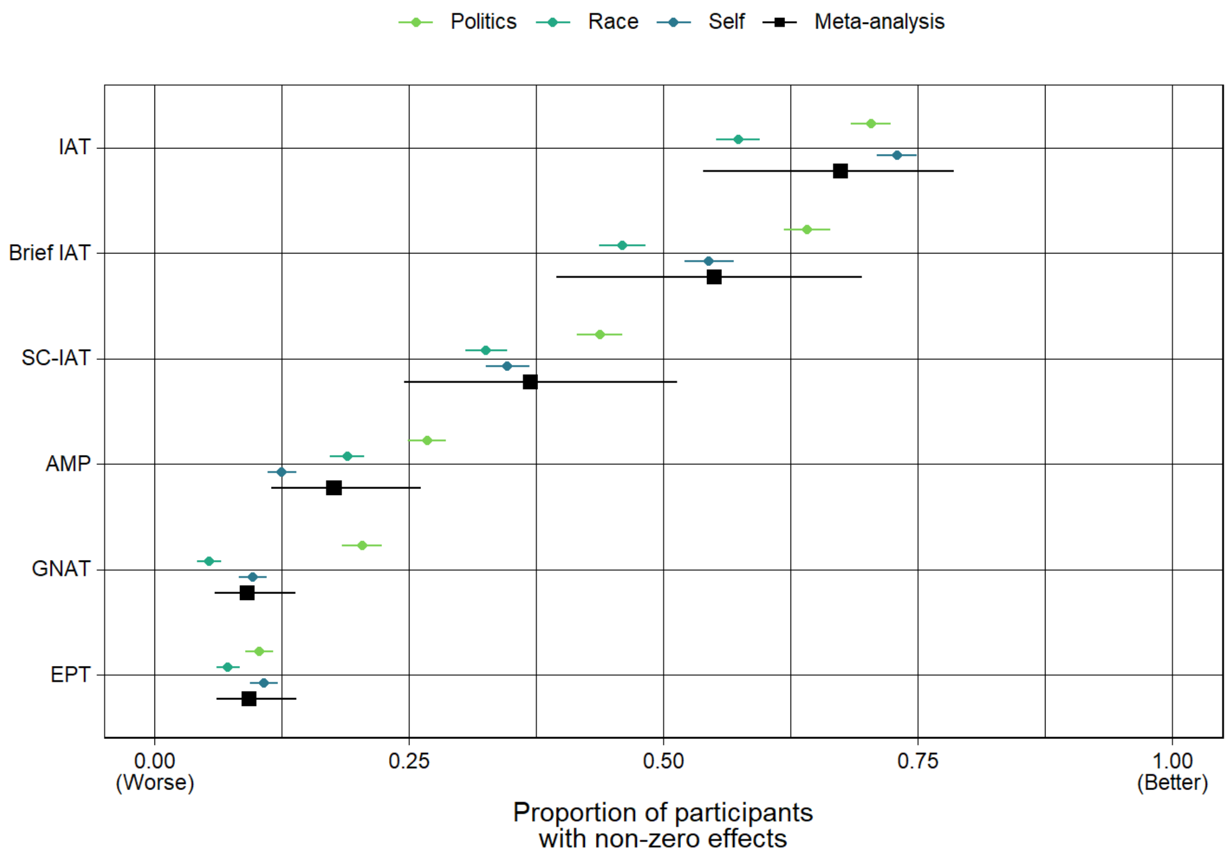

Figure S3. Plot of estimates from the logit-transformed linear mixed-effects model for RQ1.

Table S4. Results from the logit-transformed linear mixed-effects model for RQ1.

measure	estimate	ci_lower	ci_upper	pi_lower	pi_upper
IAT	0.67	0.54	0.79	0.47	0.83
Brief IAT	0.55	0.40	0.70	0.34	0.75
SC-IAT	0.37	0.24	0.51	0.20	0.58
AMP	0.18	0.11	0.26	0.09	0.32
GNAT	0.09	0.06	0.14	0.04	0.18
EPT	0.09	0.06	0.14	0.05	0.18

Table S5. Pairwise comparisons of the estimated marginal means from the logit-transformed linear mixed-effects model for RQ1.

comparison	p.value
IAT - Brief IAT	0.256
IAT - (SC-IAT)	< .001
IAT - AMP	< .001
IAT - GNAT	< .001
IAT - EPT	< .001
Brief IAT - (SC-IAT)	0.115
Brief IAT - AMP	< .001
Brief IAT - GNAT	< .001
Brief IAT - EPT	< .001
(SC-IAT) - AMP	0.005
(SC-IAT) - GNAT	< .001
(SC-IAT) - EPT	< .001
AMP - GNAT	0.008
AMP - EPT	0.008
GNAT - EPT	0.917

RQ2. Proportion of scores discriminable from other scores

Meta-analytic model

The individual level proportions were entered into a similar logit-transformed linear mixed-effects model to the previous one:

proportion_discriminable_logit ~ 1 + measure + (1 | domain),

weights = 1/variance

A forest plot of the individual effect sizes for each domain and the meta-analyzed effect size for each measure can be found in Figure S4. Data corresponding to the plot in Figure S4 can be found in Table S6. Table S7 contains the estimates for the pairwise comparisons of the estimated marginal means.

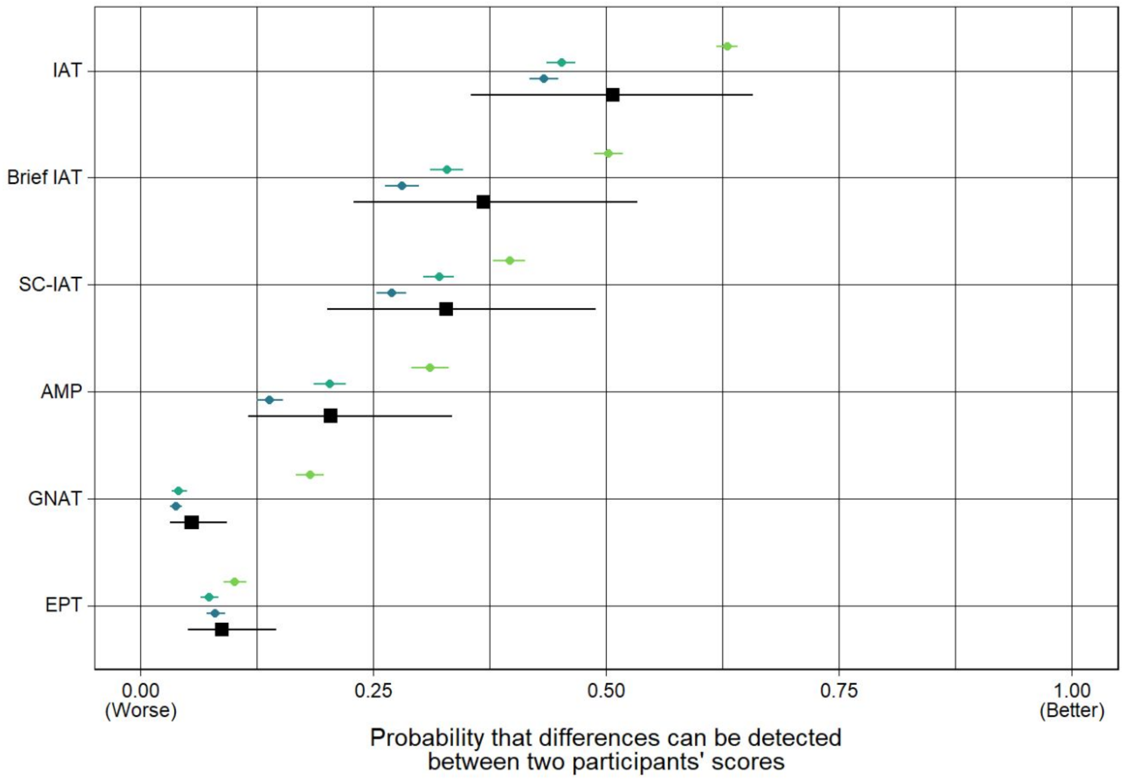

Figure S4. Plot of the estimated mean probabilities of difference between two participants' scores using the logit-transformed meta-analytic model.

Table S6. Coefficients from the meta-analytic model for RQ2.

measure	estimate	ci_lower	ci_upper	pi_lower	pi_upper
IAT	0.51	0.36	0.66	0.26	0.75
Brief IAT	0.37	0.23	0.53	0.16	0.64
SC-IAT	0.33	0.20	0.49	0.14	0.60
AMP	0.20	0.12	0.33	0.08	0.44
GNAT	0.05	0.03	0.09	0.02	0.14
EPT	0.09	0.05	0.15	0.03	0.22

-view Only

Table S7. Pairwise comparisons of the estimated marginal means for RQ2.

**comparison**	**p.value**
IAT - Brief IAT	0.115
IAT - (SC-IAT)	0.047
IAT - AMP	< .001
IAT - GNAT	< .001
IAT - EPT	< .001
Brief IAT - (SC-IAT)	0.58
Brief IAT - AMP	0.047
Brief IAT - GNAT	< .001
Brief IAT - EPT	< .001
(SC-IAT) - AMP	0.115
(SC-IAT) - GNAT	< .001
(SC-IAT) - EPT	< .001
AMP - GNAT	< .001
AMP - EPT	0.001
GNAT - EPT	0.03

For Review Only

RQ3. Coverage of Individuals' Confidence Intervals

Meta-analytic model

The proportions were entered into a similar logit-transformed linear mixed-effects model to the previous two:

ci_width_proportion_mean_logit ~ 1 + measure + (1 | domain),

weights = 1/variance

A forest plot of the individual effect sizes for each domain and the meta-analyzed effect size for each measure can be found in Figure S5. Table S8 contains the data plotted in Figure S5. Table S9 reports the pairwise comparisons of the estimated marginal means.

For Review Only

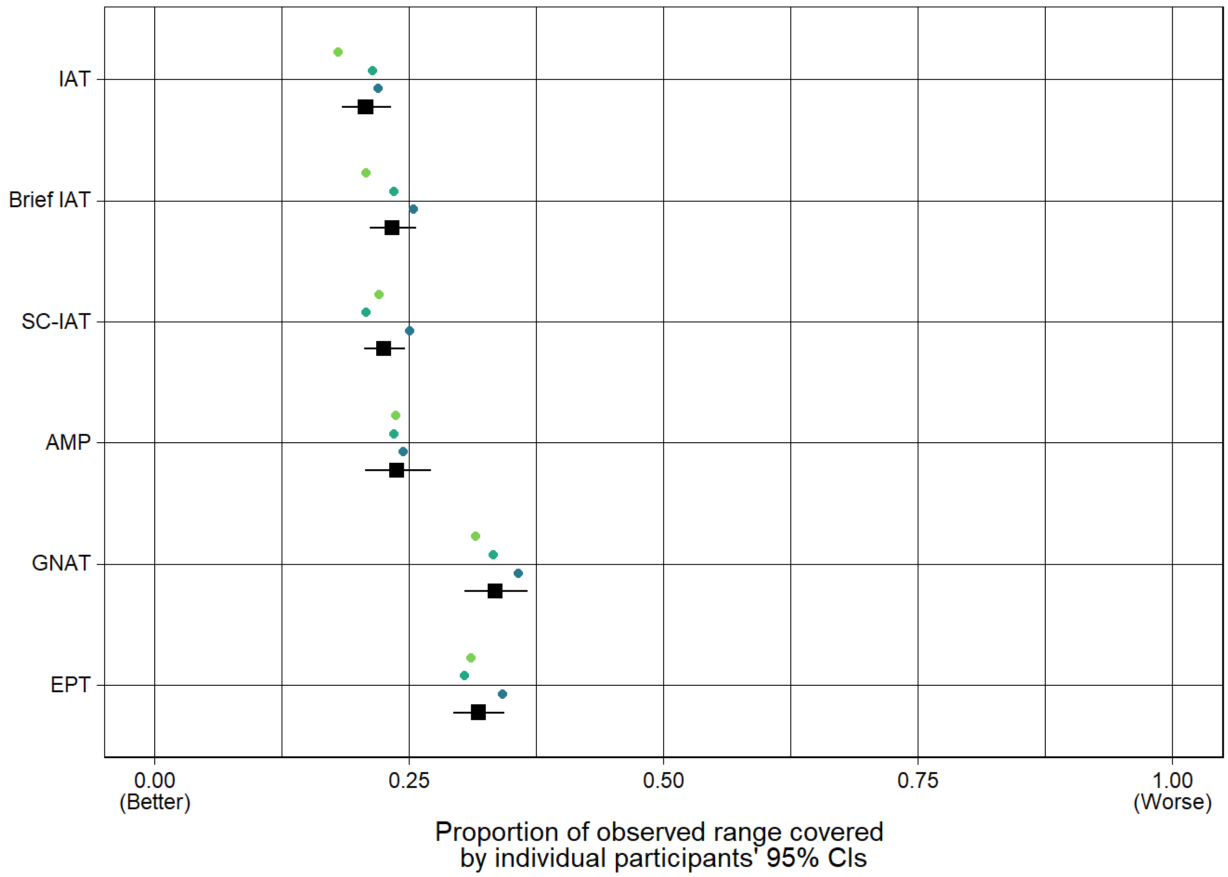

Figure S5. Plot of the estimated mean coverage of participants' scores using the logit-transformed meta-analytic model.

Table S8. Coefficients from the meta-analytic model for RQ3.

measure	estimate	ci_lower	ci_upper	pi_lower	pi_upper
IAT	0.21	0.18	0.23	0.17	0.25
Brief IAT	0.23	0.21	0.26	0.20	0.28
SC-IAT	0.23	0.21	0.25	0.19	0.27
AMP	0.24	0.21	0.27	0.19	0.29
GNAT	0.33	0.30	0.37	0.29	0.39
EPT	0.32	0.29	0.34	0.27	0.37

w Only

Table S9. Pairwise comparisons of the estimated marginal means for RQ3.

**comparison**	**p.value**
IAT - Brief IAT	0.121
IAT - (SC-IAT)	0.32
IAT - AMP	0.32
IAT - GNAT	< .001
IAT - EPT	< .001
Brief IAT - (SC-IAT)	0.876
Brief IAT - AMP	0.876
Brief IAT - GNAT	< .001
Brief IAT - EPT	< .001
(SC-IAT) - AMP	0.876
(SC-IAT) - GNAT	< .001
(SC-IAT) - EPT	< .001
AMP - GNAT	< .001
AMP - EPT	< .001
GNAT - EPT	0.582

For Review Only

Analyses conducted on explicit measures

One reviewer suggest that we computed bootstrapped confidence intervals for the three explicit measures which were collected in the dataset we used: the Modern Racism Scale (MRS), the Rosenburg Self-Esteem Scale (RSE), and the Right-Wing Authoritarianism Scale (RWA). The reviewer suggested that precision for these explicit measures may be useful to calibrate the degree of precision that one might expect from implicit measures. Although we agree calibrating implicit measures against these precision metrics could be useful, we note that such scales are not typically interpreted in an individual-level manner in the same way that implicit measures are desired to be; likewise, these measures have a much lower number of items, so direct comparison between them with implicit measures (which have a substantially greater number of items/trials) is not a true like-with-like comparison. However, if these measures were matched in terms of the number of items presented, this could indeed provide researchers with a standard of comparison in principle. The analyses for RQ1 (i.e., difference from zero point) were not meaningful to conduct with these scales, given that they have no notional zero point. We report analyses below for the equivalent of RQ2 (discriminability) and RQ3 (coverage).

CI distribution

The general distribution of CIs for each explicit measure can be found below in Figure S6.

For Review Only

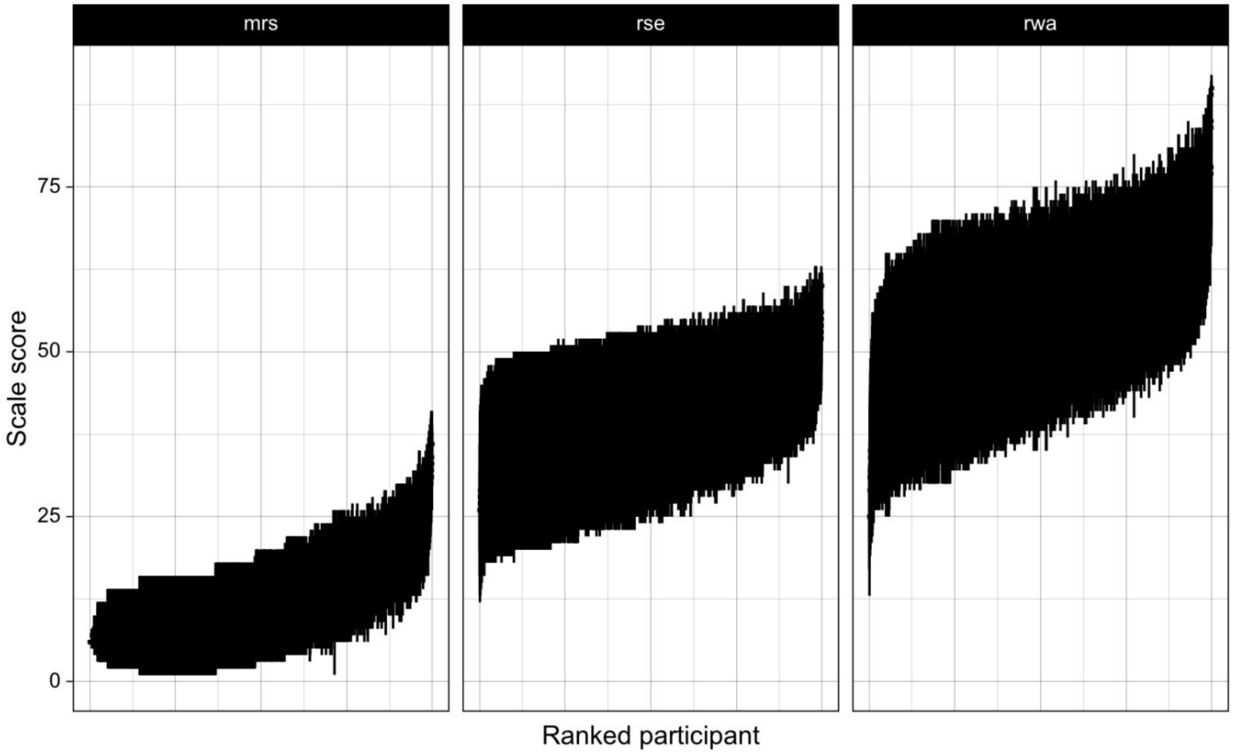

Figure S6. Caterpillar plot of the distribution of the sum scores of each explicit measure, and their associated confidence intervals, for each participant.

Explicit discriminability

We estimated discriminability of the explicit measures in a similar manner to the method employed for the implicit measures in RQ2, with one exception: our analysis was strictly fixed-effects, given that there was no possible random effect of domain for each scale. The results for this can be found below in Table S10.

Table S10. Estimates of the proportion of participants discriminable from one another in each of the three explicit measures.

scale	estimate	ci_lower	ci_upper
MRS	0.44	0.42	0.45
RSE	0.48	0.47	0.49
RWA	0.48	0.47	0.49

Explicit Coverage

We likewise estimated coverafge of the explicit measures in a similar manner to the method employed for the implicit measures in RQ3, but now modelled in a strictly fixed-effects model. The results for this can be found below in Table S11.

Table S11. Estimates of the modal coverage of the scale range from a given participants' CIs.

scale	estimate	ci_lower	ci_upper
MRS	0.17	0.17	0.17
RSE	0.19	0.19	0.19
RWA	0.20	0.20	0.20

For Review Only
